# Supplementary material for: A protein kinase coordinates cycles of autophagy and glutaminolysis in invasive hyphae of the fungus Magnaporthe oryzae within rice cells
Source: Nat Commun. 2023 Jul 12;14:4146. doi: 10.1038/s41467-023-39880-w (PMC10338429; doi:10.1038/s41467-023-39880-w)
Supplement: Supplementary file 1 — Supplementary Information [file 41467_2023_39880_MOESM1_ESM.pdf]

Supplementary Information

A protein kinase coordinates cycles of autophagy and glutaminolysis in invasive hyphae of the fungus *Magnaporthe oryzae* within rice cells

Gang Li[1,*], Ziwen Gong[1,2,*], Nawaraj Dulal[1], Margarita Marroquin-Guzman[1,3], Raquel O. Rocha[1,4], Michael Richter[1] and Richard A. Wilson[1,#]

[1]Department of Plant Pathology, University of Nebraska-Lincoln, Lincoln, NE, USA

[2]State Key Laboratory for Biology of Plant Diseases and Insect Pests, Institute of Plant Protection, Chinese Academy of Agricultural Sciences, Beijing, China

[3]Present address: Bayer CropScience, Chesterfield, MO, USA

[4]Present address: Department of Plant Pathology and Ecology, The Connecticut Agricultural Experiment Station, New Haven, CT, USA

*These authors contributed equally to this work

[#] Correspondence to: rwilson10@unl.edu

This file contains:

- Supplementary Figures
- Supplementary Figure Legends
- Supplementary References

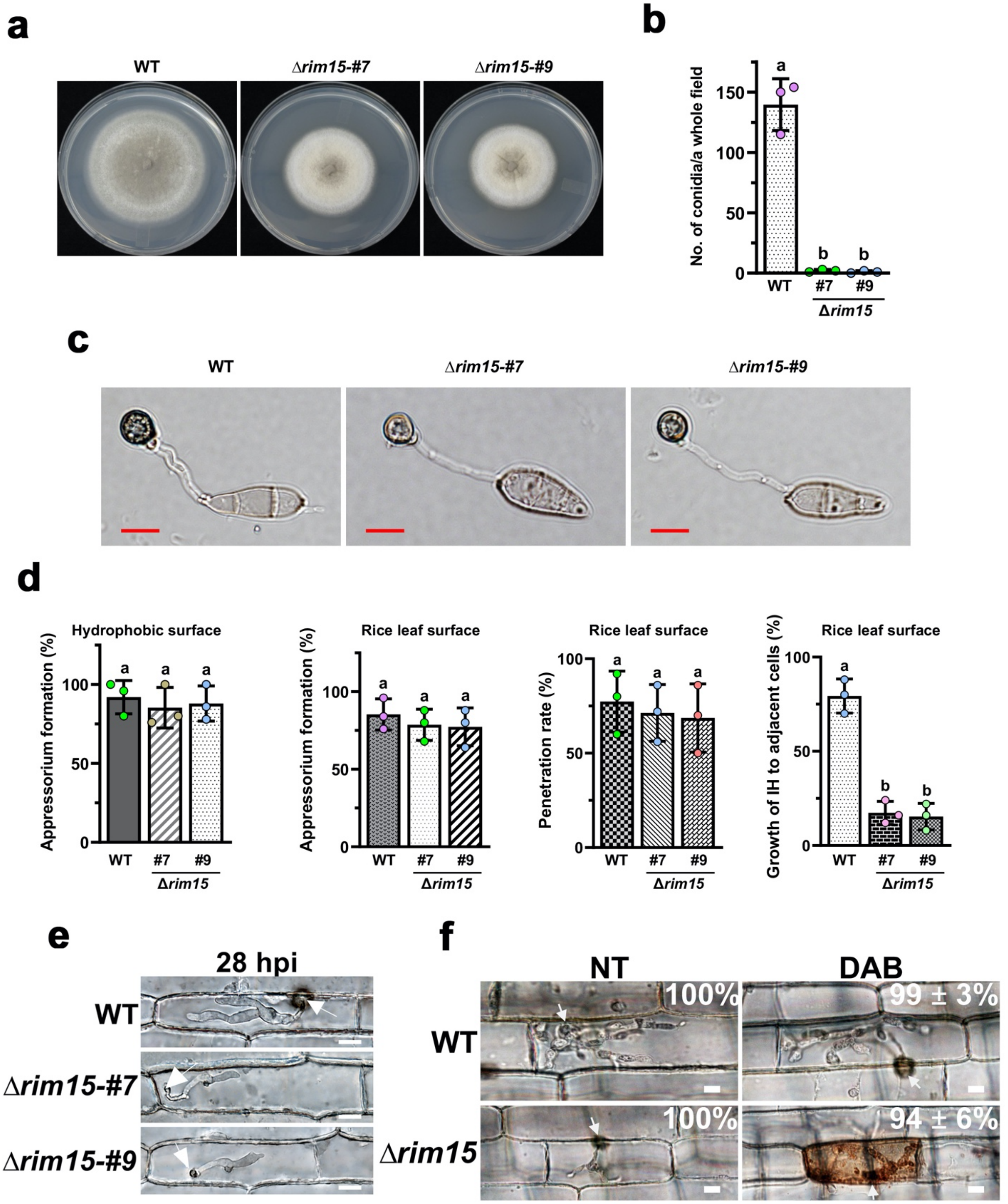

Supplementary Figure 1. ***RIM15*** **is required for invasive hyphae cell-to-cell growth and for suppressing the host ROS burst. a.** Plate test showing how on complete media (CM), the radial growth

of two independent Δ*rim15* gene knockout mutants was reduced compared to WT. **b.** Sporulation rates of two independent Δ*rim15* mutants were reduced on CM compared to WT. Spores of the indicated strains were harvested from CM at 10 days, and the number of spores was counted using a haemocytometer under a dissecting microscope. Data are presented as mean values ± SD. n = 3 biological replicates. For each column, different letters indicate significant differences of the means at $P \leq 0.05$. *P*-values were determined by Welch ANOVA with Games-Howell post hoc test. $P = 0.0144$ between WT and Δ*rim15-#7*, $P = 0.0142$ between WT and Δ*rim15-#9*, and $P = 0.5015$ between Δ*rim15-#7* and Δ*rim15-#9*. **c.** Loss of *RIM15* did not impair appressorium formation on artificial hydrophobic surfaces. Spores were harvested from 14-day-old colonies of WT and two Δ*rim15* mutant strains growing on oatmeal agar medium and then resuspended at $2x10^4$ spores ml^{-1}. 200 µl of the spore suspension was inoculated onto a hydrophobic plastic cover slip positioned in a humid compartment and then incubated at 25°C for 24 hr in the dark. Scale bar is 10 µm. Experiments were repeated in triplicate. **d.** Graphs showing how appressorium formation rates on artificial hydrophobic surfaces (plastic coverslips) and on detached rice leaf sheath surfaces at 24 hours post inoculation (hpi), and appressorial penetration rates on detached rice leaf sheath surfaces at 30 hpi, were not significantly different ($P \leq 0.05$) between Δ*rim15* mutant strains and WT. However, cell-to-cell movement rates in detached rice leaf sheath epidermal cells at 48 hpi were significantly ($P \leq 0.05$) reduced in two independent Δ*rim15* mutant strains compared to WT. For appressorium formation and penetration rates, 50 spores or appressoria, respectively, were observed for each strain per coverslip or detached rice leaf sheath. For IH movement rates, 50 primary infected epidermal cells were observed for each strain per leaf sheath. Values are means ± SD. n = 3 biological replicates. For each column, different letters indicate significant differences of the means at $P \leq 0.05$. *P*-values were determined by one-way ANOVA with Tukey's multiple comparisons test. For appressorium formation rates on artificial hydrophobic surfaces, $P = 0.769$ between WT and Δ*rim15-#7*, $P = 0.907$ between WT and Δ*rim15-#9*, and $P = 0.957$ between Δ*rim15-#7* and Δ*rim15-#9*. For appressorium formation rates on rice leaf surfaces, $P = 0.743$ between WT and Δ*rim15-#7*, $P = 0.657$ between WT and Δ*rim15-#9*, and $P = 0.988$ between Δ*rim15-#7* and Δ*rim15-#9*. For appressorium penetration rates on rice leaf surfaces, $P = 0.898$ between WT and Δ*rim15-#7*, $P = 0.802$ between WT and Δ*rim15-#9*, and $P =$ 0.979 between Δ*rim15-#7* and Δ*rim15-#9*. For cell-to-cell movement of IH between rice cells, $P = 0.001$ between WT and Δ*rim15-#7*, $P =$ 0. 0.001 between WT and Δ*rim15-#9*, and $P = 0.9432$ between Δ*rim15-#7* and Δ*rim15-#9*. **e.** Live-cell imaging at 28 hpi of detached rice leaf sheath epidermal cells infected with the indicated strains showing how Δ*rim15* mutant strains are impaired for biotrophic growth. **f.** Live cell-imaging of detached rice leaf sheaths infected with the indicated strains shows how loss of *RIM15* elicits an oxidative burst in Δ*rim15*-infected host rice cells. Leaf sheaths were stained with 3,3′-Diaminobenzidine (DAB) and imaged at 36 hpi. NT is no treatment. **e,f.** White arrows indicate appressorial penetration sites. Scale bar is 10 µm. Representative images and values are derived from

observing 50 infected rice cells per leaf sheath per treatment. n = 3 biological replicates. Values are means ± SD. **b,d,f.** Source data are provided as a Source Data file.

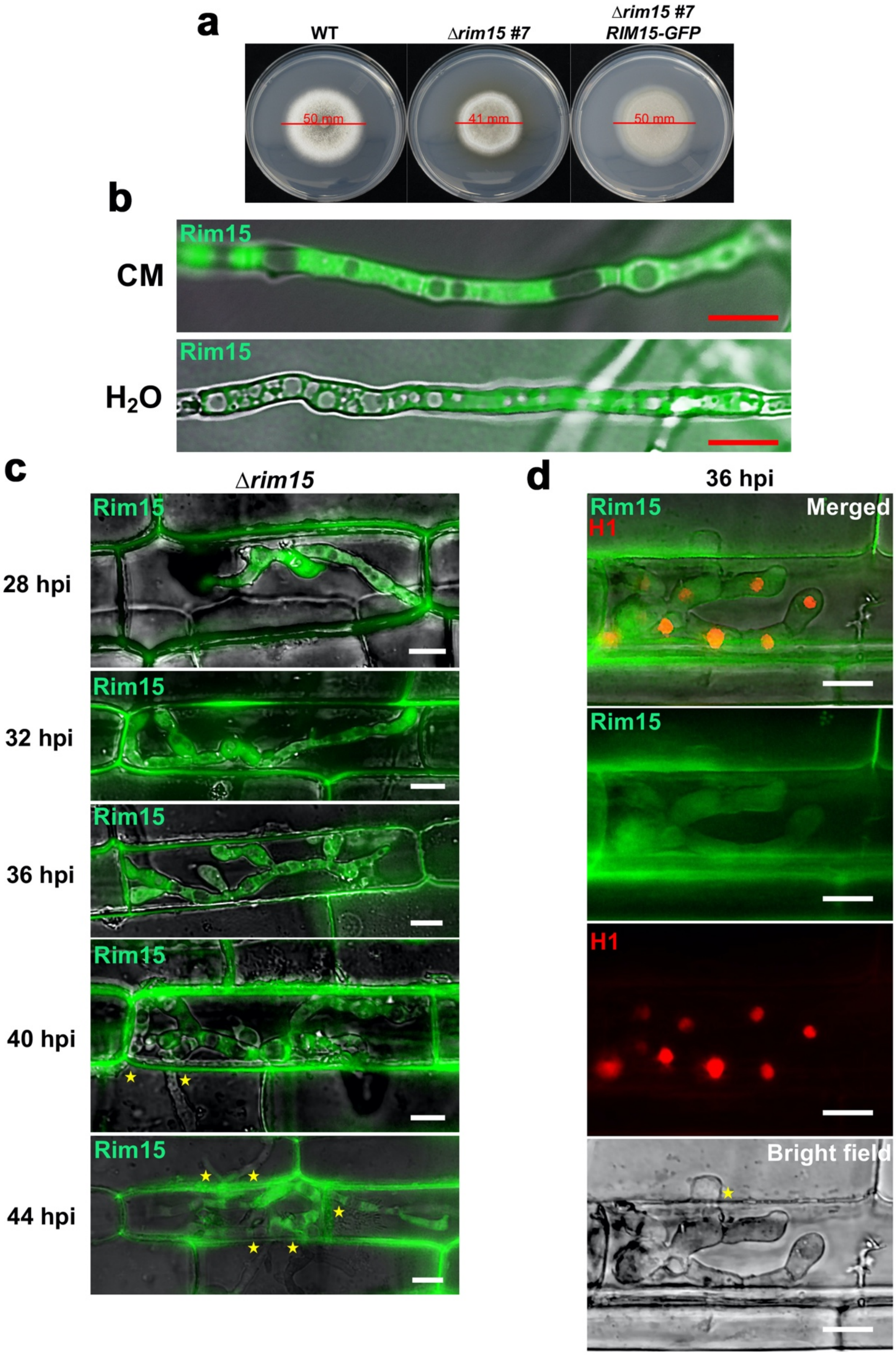

Supplementary Figure 2. Rim15 localizes to hyphal cytoplasm. a. Complementation of Δ*rim15* with *RIM15-GFP* restored radial growth on CM media, thus *RIM15-GFP* is functional in Δ*rim15*. **b.** Micrographs

showing that in vegetative hyphae, unlike in yeast, *M. oryzae* Rim15-GFP remains cytoplasmic under both nutrient-rich (CM) and nutrient-starvation (H_2O) culture shake conditions. Here, the Δ*rim15 RIM15-GFP* complementation strain was grown in liquid CM for 42 hr. After washing with water, vegetative hyphae were transferred into fresh liquid CM or water for a further 3.5 hr before imaging. Bar is 10 µm. Merged channel is shown. **c.** Live-cell imaging at the indicated times of detached rice leaf sheath epidermal cells infected with the Δ*rim15 RIM15-GFP* complementation strain shows that Rim15-GFP localizes to IH cytoplasm throughout biotrophy. Asterisks indicate movement of IH into neighbouring cells. Bar is 10 µm. Merged channel is shown. **d.** Live-cell imaging at 36 hpi of detached rice leaf sheath epidermal cells infected with a *RIM15*$^+$ strain expressing histone H1-RFP and Rim15-GFP shows that Rim15-GFP does not co-localize with H1-RFP in the nucleus. Asterisks indicate movement of IH into neighbouring cells. Bar is 10 µm. **b-d,** Representative images are derived from observing 50 infected rice cells per leaf sheath per treatment. n = 3 biological replicates.

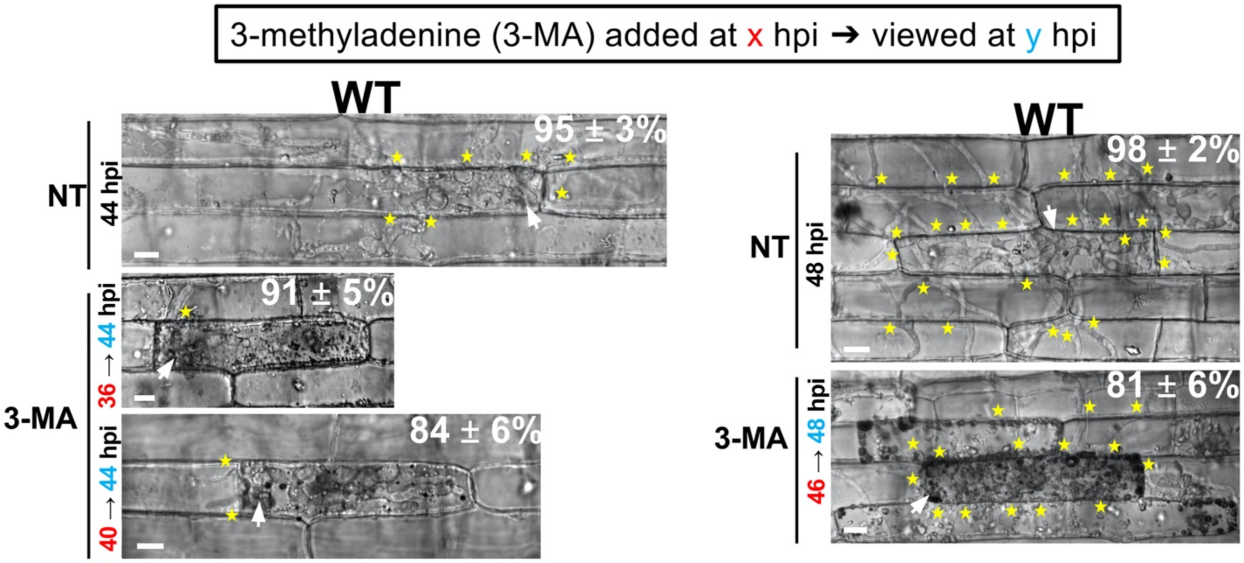

Supplementary Figure 3. Inhibiting autophagic cycling during biotrophy abolishes IH growth. Live-cell imaging of WT-infected detached rice leaf sheath epidermal cells shows that, when viewed at the indicated times following treatment with 10 mM 3-methlyadenine (3-MA) in 1 % DMSO, and when compared to the untreated controls (1 % DMSO), inhibiting autophagy prevented further IH growth. White arrows indicate appressorial penetration sites. Asterisks indicate movement of IH into neighbouring cells. NT is no treatment. Bar is 10 µm. Bright field channel is shown. Representative images and values are derived from observing 50 infected rice cells per leaf sheath per treatment. n = 3 biological replicates. Values are means ± SD. Source data are provided as a Source Data file.

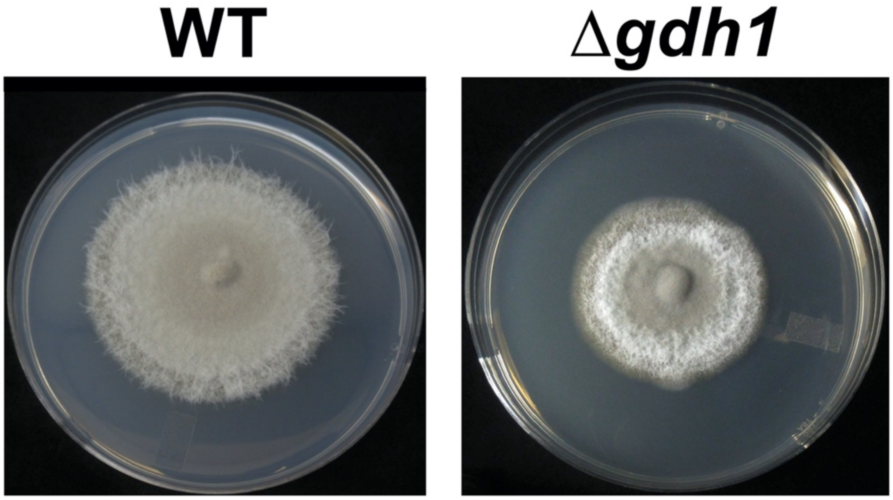

Supplementary Figure 4. Gdh1 is required for optimal growth on NH_4^+-containing media. The indicated strains were grown on plates of minimal media with 1 % (w/v) glucose as the sole carbon source and 5 mM ammonium tartrate (ie 10 mM NH_4^+) as the sole nitrogen source. Images were taken after 10 days growth.

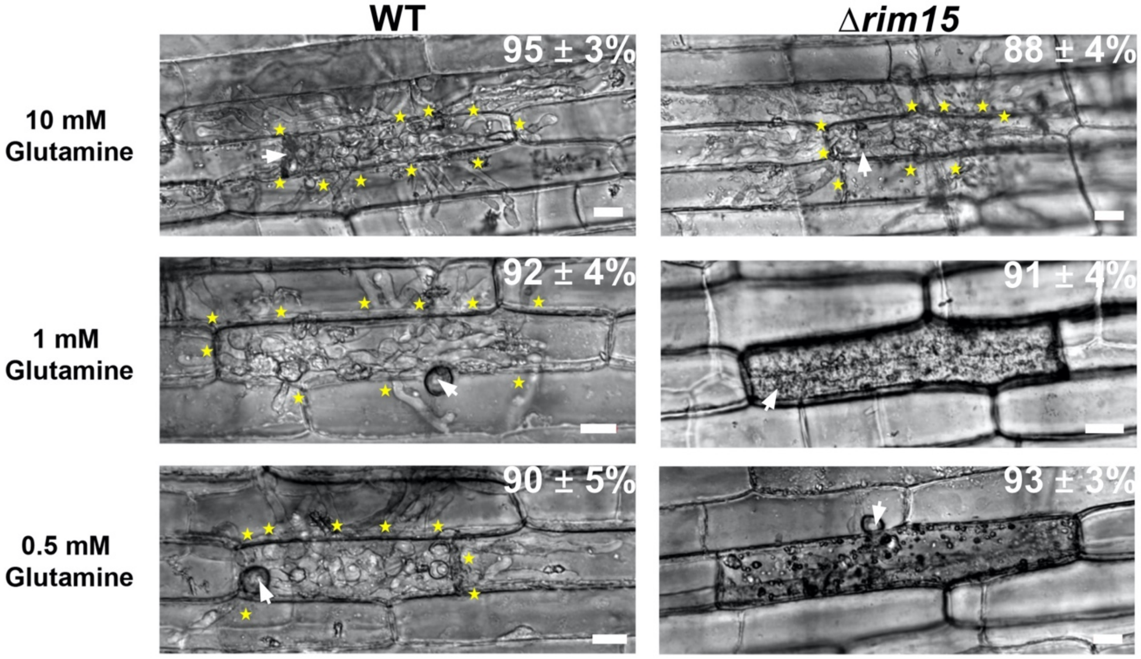

Supplementary Figure 5. Remediation of Δ*rim15* biotrophic growth by glutamine is concentration dependent. Live-cell imaging at 44 hpi of detached rice leaf sheath epidermal cells infected with the indicated strains shows that glutamine treatment at 36 hpi did not remediate Δ*rim15* biotrophic growth when added at concentrations < 10 mM. White arrows indicate appressorial penetration sites. Asterisks indicate movement of IH into neighbouring cells. Bar is 10 μm. Bright field channel is shown. Representative images and values are derived from observing 50 infected rice cells per leaf sheath per treatment. n = 3 biological replicates. Values are means ± SD. Source data are provided as a Source Data file.

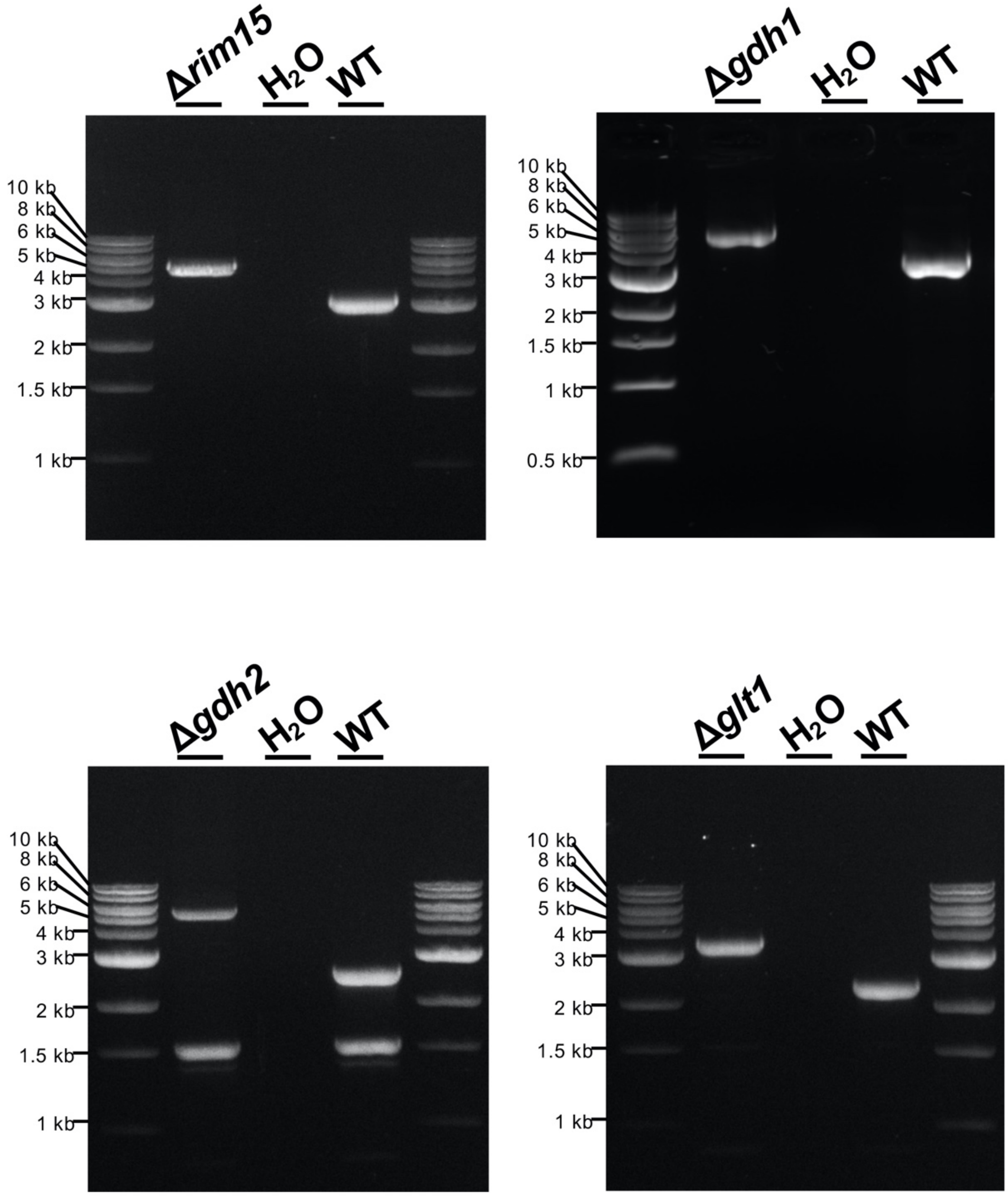

Supplementary Figure 6. Molecular identification of gene knockout mutants. For *RIM15* (*top left*) and *GDH1* (*top right*) gene deletions, 1 kb of the respective gene coding region beginning with the start codon was replaced by the 2.8 kb sulphonylurea resistance gene *ILV1*[1]. To identify gene deletants, PCR primers designed outside the region of homologous recombination were used to amplify, from putative knockout mutant genomic DNA, a 4.8 kb DNA fragment comprising 1 kb of upstream sequence, the 2.8 kb *ILV1* gene, and 1 kb of downstream sequence. The same primer pairs amplified a 3 kb DNA fragment from heterologous transformant or WT genomic DNA containing 1 kb of the upstream sequence, 1 kb of the gene coding region, and 1 kb of the downstream sequence.

For the *GDH2* gene deletion (*bottom left*), 0.5 kb of the gene coding region from the start codon was replaced by the sulphonylurea resistance gene *ILV1*. For identification of Δ*gdh2* deletants, a 4.8 kb DNA fragment, containing 1 kb of upstream sequence, the 2.8 kb *ILV1* gene, and 1 kb of downstream sequence, was amplified from the genomic DNA of positive transformants. Using the same primer pairs, amplification from the DNA of heterologous transformants and WT produced a 2.5 kb DNA fragment containing 1 kb of upstream sequence, 0.5 kb of the gene coding region, and 1 kb of downstream sequence.

For the *GLT1* gene deletion (*bottom right*), 0.5 kb of the gene coding region from the start codon was replaced by the hygromycin resistance gene *hph*[1]. For identification of gene deletants, a 3.5 kb DNA fragment, containing 1 kb of upstream sequence, the 1.5 kb *hph* gene, and 1 kb of downstream sequence, was amplified from genomic DNA of the transformants. Heterologous transformants and WT yielded instead a 2.5 kb DNA fragment containing 1 kb of upstream sequence, 0.5 kb of the gene coding region, and 1 kb of downstream sequence.

Each set of PCR experiments were independently performed twice, with similar results.

Supplementary References

1. Wilson, R. A., Gibson, R. P., Quispe, C. F., Littlechild, J. A. & Talbot, N. J. An NADPH-dependent genetic switch regulates plant infection by the rice blast fungus. *Proc. Natl. Acad. Sci. U. S. A*. **107,** 21902-21907 (2010).
