# Supplementary material for: A protein kinase coordinates cycles of autophagy and glutaminolysis in invasive hyphae of the fungus Magnaporthe oryzae within rice cells
Source: Nat Commun. 2023 Jul 12;14:4146. doi: 10.1038/s41467-023-39880-w (PMC10338429; doi:10.1038/s41467-023-39880-w)
Supplement: Supplementary file 3 — Description of Additional Supplementary Files [file 41467_2023_39880_MOESM3_ESM.pdf]

Description of Additional Supplementary Files

Supplementary Data 1
Description: Proteomic analysis of protein abundances in *Δrim15* and Guy11 vegetative hyphae after growth on minimal media with glutamine as the sole carbon and nitrogen source.

Supplementary Data 2
Description: Phosphoproteomic analysis of protein extracts from *Δrim15* and Guy11 vegetative hyphae after growth on minimal media with glutamine as the sole carbon and nitrogen source.

Supplementary Data 3
Description: Metabolomic analysis of *Δrim15* and Guy11 vegetative hyphae after growth on minimal media with glutamine as the sole carbon and nitrogen source.

Supplementary Data 4
Description: Metabolomic analysis of *Δrim15* and Guy11 vegetative hyphae after growth on minimal media with glutamine as the sole carbon and nitrogen source.

Supplementary Data 5
Description: Strains used in this study.

Supplementary Data 6
Description: Oligonucleotide primers used in this study.

Supplementary Data 7
Description: Plasmids used in this study.
