# Supplementary material for: A protein kinase coordinates cycles of autophagy and glutaminolysis in invasive hyphae of the fungus Magnaporthe oryzae within rice cells
Source: Nat Commun. 2023 Jul 12;14:4146. doi: 10.1038/s41467-023-39880-w (PMC10338429; doi:10.1038/s41467-023-39880-w)
Supplement: Supplementary file 11 — Reporting Summary [file 41467_2023_39880_MOESM11_ESM.pdf]

nature portfolio

Corresponding author(s): Richard A. Wilson

Last updated by author(s): Jun 19, 2023

Reporting Summary

Nature Portfolio wishes to improve the reproducibility of the work that we publish. This form provides structure for consistency and transparency in reporting. For further information on Nature Portfolio policies, see our Editorial Policies and the Editorial Policy Checklist.

Statistics

For all statistical analyses, confirm that the following items are present in the figure legend, table legend, main text, or Methods section.

n/a	Confirmed	
☐	☒	The exact sample size (*n*) for each experimental group/condition, given as a discrete number and unit of measurement
☐	☒	A statement on whether measurements were taken from distinct samples or whether the same sample was measured repeatedly
☐	☒	The statistical test(s) used AND whether they are one- or two-sided<br>*Only common tests should be described solely by name; describe more complex techniques in the Methods section.*
☒	☐	A description of all covariates tested
☐	☒	A description of any assumptions or corrections, such as tests of normality and adjustment for multiple comparisons
☐	☒	A full description of the statistical parameters including central tendency (e.g. means) or other basic estimates (e.g. regression coefficient) AND variation (e.g. standard deviation) or associated estimates of uncertainty (e.g. confidence intervals)
☐	☒	For null hypothesis testing, the test statistic (e.g. *F*, *t*, *r*) with confidence intervals, effect sizes, degrees of freedom and *P* value noted<br>*Give P values as exact values whenever suitable.*
☒	☐	For Bayesian analysis, information on the choice of priors and Markov chain Monte Carlo settings
☒	☐	For hierarchical and complex designs, identification of the appropriate level for tests and full reporting of outcomes
☒	☐	Estimates of effect sizes (e.g. Cohen's *d*, Pearson's *r*), indicating how they were calculated

Our web collection on statistics for biologists contains articles on many of the points above.

Software and code

Policy information about availability of computer code

Data collection	Confocal images were taken by a Nikon Eclipse Ni-E upright microscope.<br>Proteomics was analyzed by nanoLC-MS/MS feeding into an Orbitrap Eclipse mass spectrometer.<br>Phosphoproteomics was achieved first by TiO2 phosphopeptide enrichment and then analyzed by nanoLC-MS/MS feeding into an Orbitrap Eclipse mass spectrometer.<br>Metabolomics was performed using MS-Dial (data-independent acquisition (DIA) in liquid chromatography (LC) coupled to tandem mass spectrometry (MS/MS)) and chromatograms were analyzed using MetaboAnalyst 5.0.<br>DNA bands were separated by agarose gel electrophoresis and imaged using ChemiDoc XRS+ system (Bio-Rad, USA) with Image Lab software (version 5.2.1, Bio-Rad).<br>The immunoblots were imaged using the Clarity™ Western ECL chemiluminescent system (Bio-Rad). Infected rice leaves were imaged using an Epson Perfection V550 scanner. Plate images were taken with a Sony Cyber-shot digital camera, 14.1 megapixels.
Data analysis	Means ± SD values for the fluorescent protein distribution patterns, vegetative hyphal growth on plates, and invasive hyphal growth in infected rice cells were calculated by Microsoft Excel 2016.<br>Comparison of the mean values of sporulation rates, appressorium formation rates, penetration rates and cell-to-cell movement rates were performed using the one-way ANOVA function in PASW Statistics 18.0 (PASW Statistics Inc.) with the Tukey HSD multiple comparison test.<br>Confocal images were processed by the software NIS-Elements (version 5.20.02).<br>For the proteomics and phosphoproteomics data, quantification was performed using Proteome Discoverer (Thermo; version 2.4), and all MS/MS samples were searched using Mascot (Matrix Science, London, UK; version 2.6.2).<br>The localization probability of the phosphorylation sites were calculated using PtmRS.<br>Metabolomic data were analyzed using MetaboAnalyst 5.0.

Phosphorylated proteins in immunoblot analysis were quantified by ImageJ (Http://imagej.nih.gov/ij; version 1.53t, Java 1.8.0_265 (64-bit)).

For manuscripts utilizing custom algorithms or software that are central to the research but not yet described in published literature, software must be made available to editors and reviewers. We strongly encourage code deposition in a community repository (e.g. GitHub). See the Nature Portfolio guidelines for submitting code & software for further information.

Data

Policy information about availability of data

All manuscripts must include a data availability statement. This statement should provide the following information, where applicable:

- Accession codes, unique identifiers, or web links for publicly available datasets
- A description of any restrictions on data availability
- For clinical datasets or third party data, please ensure that the statement adheres to our policy

All data are available in the manuscript or in Supplementary Information. The M. oryzae RIM15, GLT1, GDH1, GDH2, ATG8, SOD1, PWL2 and BAS4 gene sequences are available at NCBI under the accession numbers MGG_00345 (https://www.ncbi.nlm.nih.gov/gene/2675109), MGG_07187 (https://www.ncbi.nlm.nih.gov/gene/?term=MGG_07187), MGG_08074 (https://www.ncbi.nlm.nih.gov/gene/?term=MGG_08074), MGG_05247 (https://www.ncbi.nlm.nih.gov/gene/?term=MGG_05247), MGG_01062 (https://www.ncbi.nlm.nih.gov/gene/?term=MGG_01062), MGG_02625 (https://www.ncbi.nlm.nih.gov/gene/?term=MGG_02625), MGG_13863 (https://www.ncbi.nlm.nih.gov/gene/?term=MGG_13863) and MGG_10914 (https://www.ncbi.nlm.nih.gov/gene/?term=MGG_10914), respectively. Uniprot accessions can be accessed at https://www.uniprot.org. The proteomics data generated in this study have been deposited to the ProteomeXchange Consortium via the PRIDE partner repository (http://www.ebi.ac.uk/pride) with the dataset identifier PXD04307. Uncropped western blots are provided in Supplementary Information. Mutant strains generated in this study are available from the corresponding author with an appropriate APHIS permit. Source data underlying all mean values are provided with this paper.

Research involving human participants, their data, or biological material

Policy information about studies with human participants or human data. See also policy information about sex, gender (identity/presentation), and sexual orientation and race, ethnicity and racism.

Reporting on sex and gender	N/A
Reporting on race, ethnicity, or other socially relevant groupings	N/A
Population characteristics	N/A
Recruitment	N/A
Ethics oversight	N/A

Note that full information on the approval of the study protocol must also be provided in the manuscript.

Field-specific reporting

Please select the one below that is the best fit for your research. If you are not sure, read the appropriate sections before making your selection.

[x] Life sciences   [ ] Behavioural & social sciences   [ ] Ecological, evolutionary & environmental sciences

For a reference copy of the document with all sections, see nature.com/documents/nr-reporting-summary-flat.pdf

Life sciences study design

All studies must disclose on these points even when the disclosure is negative.

Sample size	Sample size was chosen according to long-term experience in biological and biochemical experimentation, for example following Marroquin-Guzman et al. 2017, Nature Microbiology, and Li et al. 2020, New Phytologist.<br>Pathogenicity-related assays were conducted according to published protocols.<br>For inoculation assays, each independent experiment contains at least 3 randomly selected rice leaves.<br>For analysis of the distribution of fluorescent proteins in growing fungal invasive hyphae, at least 50 invasive hyphae expressing fluorescent proteins were observed.<br>For analysis of the fluorescent proteins distribution in growing fungal invasive hyphae in infected rice cells, at least 50 infected rice cells were observed.
Data exclusions	No data were excluded from the analyses.
Replication	All experiments were performed with three biological replicates and similar results were obtained. The information has been provided in the Source Data file.
Randomization	Related fungal strains were initially grown on the same condition (complete media) and randomly allocated for each experimental test.<br>The rice seedling were grown on the same condition and randomly allocated for inoculation assay.<br>The distribution of fluorescent proteins in fungal invasive hyphae were observed in randomly selected infected rice cells.

	Biological samples used in proteomics, phosphoproteomics, and metabolomics analyses were all selected by random sampling.
Blinding	Investigators were not blinded to group allocation during the experiments, but data collection and assessment were repeated by multiple independent experiments to ensure that the analysis is as objective as possible.

Reporting for specific materials, systems and methods

We require information from authors about some types of materials, experimental systems and methods used in many studies. Here, indicate whether each material, system or method listed is relevant to your study. If you are not sure if a list item applies to your research, read the appropriate section before selecting a response.

Materials & experimental systems

n/a	Involved in the study
☐	☒ Antibodies
☒	☐ Eukaryotic cell lines
☒	☐ Palaeontology and archaeology
☒	☐ Animals and other organisms
☒	☐ Clinical data
☒	☐ Dual use research of concern
☒	☐ Plants

Methods

n/a	Involved in the study
☒	☐ ChIP-seq
☒	☐ Flow cytometry
☒	☐ MRI-based neuroimaging

Antibodies

Antibodies used	S6K phospho-status analysis: Anti-phospho-p70 S6 kinase α antibody (monoclonal, A-6, produced in mouse, Santa Cruz Biotechnology, #sc-8416, USA);<br>Anti-Tub(α) antibody (monoclonal, YOL1/34, produced in rat, Santa Cruz Biotechnology, #sc-53030, USA);<br>Autophagy monitoring: Anti-Green Fluorescent Protein (GFP) antibody (monoclonal, GSN149, produced in mouse, Sigma, #G1546, USA);<br>Anti-mouse IgG-peroxidase (produced in goat, Sigma, #A2304, USA);<br>Anti-rat IgG-peroxidase (produced in goat, Santa Cruz Biotechnology, #sc-2006,USA).
Validation	All antibodies used in this paper are commercially available and widely used in the scientific community. The following antibodies were certified and validated by the supplier:<br>Anti-phospho-p70 S6 kinase α antibody: https://www.scbt.com/p/p-p70-s6-kinase-alpha-antibody-a-6<br>Anti-Tub(α) antibody: https://www.scbt.com/p/alpha-tubulin-antibody-yol1-34<br>Anti-Green Fluorescent Protein (GFP) antibody: https://www.sigmaaldrich.com/US/en/product/sigma/g1546
